# Williams-Beuren's Syndrome: A Case Report

**DOI:** 10.1155/2012/585726

**Published:** 2012-08-13

**Authors:** Hassan Zamani, Kazem Babazadeh, Saeid Fattahi, Farzad Mokhtari-Esbuie

**Affiliations:** ^1^Non-Communicable Pediatric Diseases Research Center, Babol University of Medical Sciences, Mazandaran, Iran; ^2^Mazandaran University of Medical Sciences, Sari, Iran; ^3^Fatemeh Zahra Hospital, Mazandaran University of Medical Science, Sari, Iran

## Abstract

Williams-Beuren syndrome is a rare familial multisystem disorder occurring in 1 per 20,000 live births. It is characterized by congenital heart defects (CHD), skeletal and renal anomalies, cognitive disorder, social personality disorder and dysmorphic facies. We present a case of Williams syndrome that presented to us with heart murmur and cognitive problem. A 5-year-old girl referred to pediatric cardiologist because of heart murmurs. She had a systolic murmur (2-3/6) in right upper sternal border with radiation to right cervical region. She also had a bulge forehead. Angiography showed mild supra valvular aortic stenosis and mild multiple peripheral pulmonary stenosis. Fluorescent in situ hybridization (FISH) was performed and the result was: 46.XX, ish del (7q11.2) (ELN X1) (7q22 X2) ELN deletion compatible with Williams syndrome. Peripheral pulmonary artery stenosis is associated with Noonan syndrome, Alagille syndrome, Cutis laxa, Ehler-Danlos syndrome, and Silver-Russel syndrome. The patient had peripheral pulmonary artery stenosis, but no other signs of these syndromes were present, and also she had a supravalvular aortic stenosis which was not seen in other syndromes except Williams syndrome. *Conclusion*. According to primary symptoms, paraclinical and clinical finding such as dysmorphic facies, cognitive disorder and congenital heart defect, Williams syndrome was the first diagnosis. We suggest a more attention for evaluating heart murmur in childhood period, especially when the patient has abnormal facial features or mental problem.

## 1. Introduction

Williams-Beuren's syndrome is a rare familial multisystem disorder that occurs in 1 per 20,000 live births. It is characterized by congenital heart defects (CHD), neonatal hypercalcemia, skeletal and renal anomalies, cognitive disorder, social personality disorder, and dysmorphic facies [1]. The neurocognitive profile of Williams' syndrome most commonly includes mild mental retardation. Cognitive strengths and weaknesses relative to other patients with mental retardation include relatively good auditory rote memory but extreme difficulty with visuospatial construction tasks [2]. Most of patients with Williams' syndrome have CHDs, which typically include supravalvar aortic stenosis and/or supravalvular pulmonary stenosis [3, 4]. Patient with Williams' syndrome may commonly develop hypertension either because of renal artery stenosis or other undefined etiologies [3]. Approximately 90% of patients with the clinical diagnosis of Williams' syndrome have a deletion at chromosome 7q11.23, which can be detected by FISH (fluorescent in situ hybridization). The genes mapping to this region have been defined and include the elastin gene, ELN. Mutations within the elastin gene have also been found in patients with isolated supravalvar aortic stenosis [5, 6], and deletion of the elastin gene in patients with Williams' syndrome is thought to account for the cardiovascular phenotype [7].

## 2. Case Presentation

**Figure 1:**
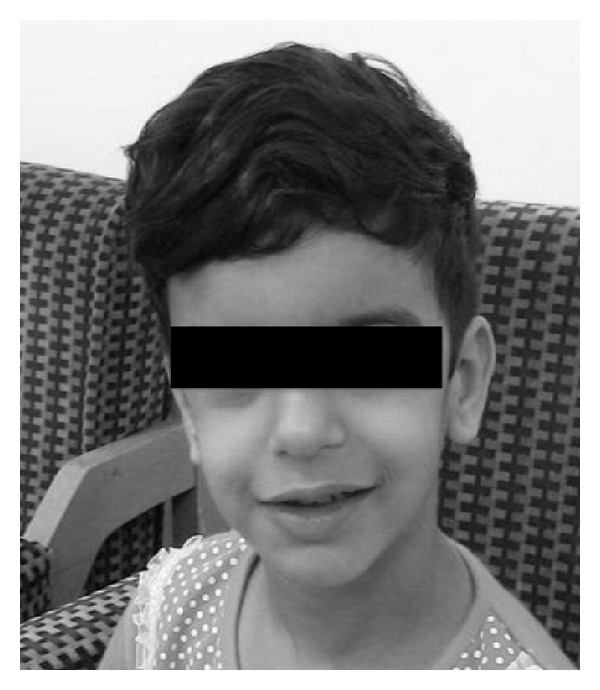
Elfin faces.

**Figure 2:**
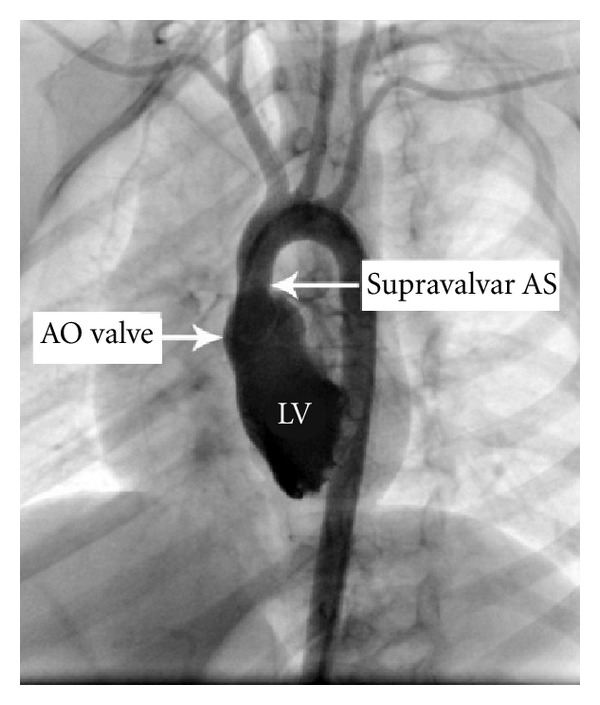
Angiography. Left ventricle injection: supraaortic stenosis.

**Figure 3:**
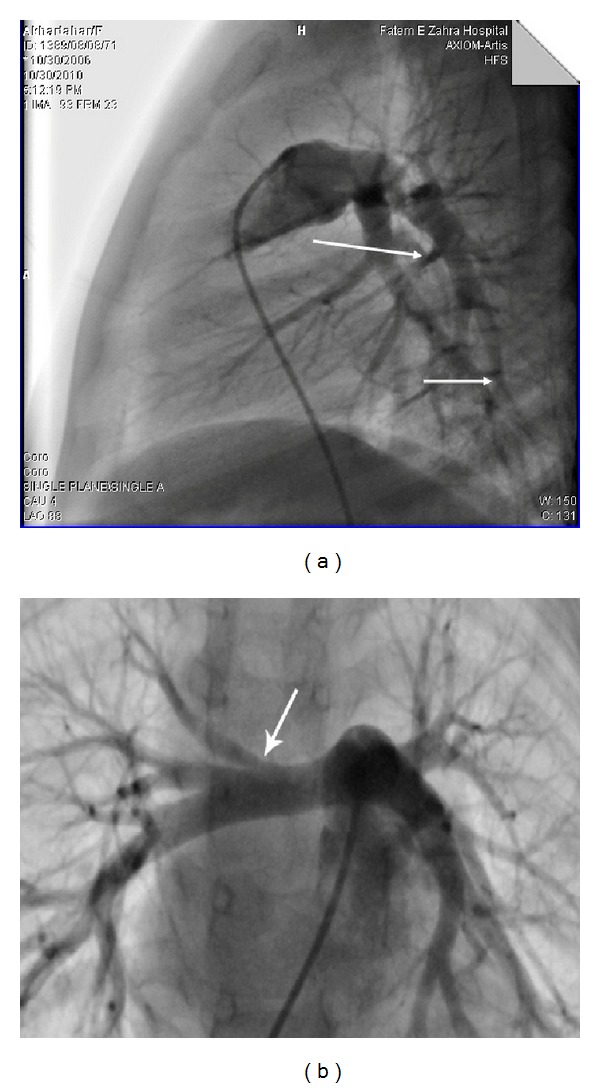
Angiography. Pulmonary artery injection: peripheral pulmonary stenosis.

**Figure 4:**
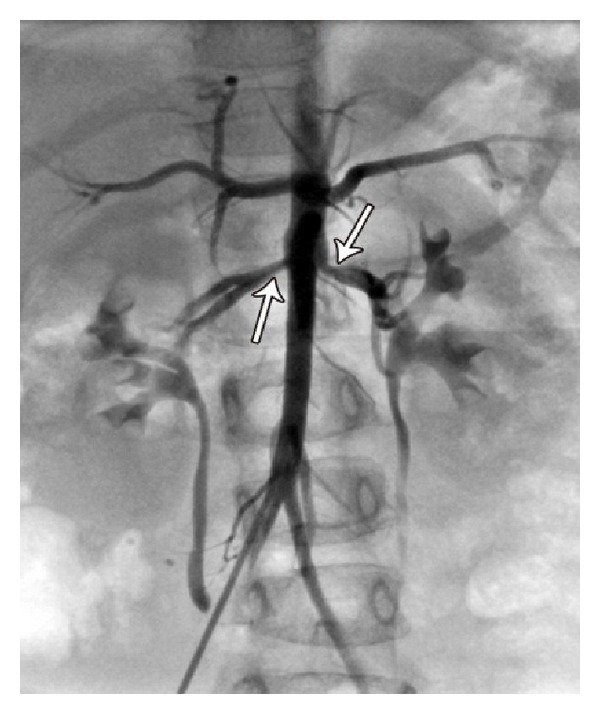
Angiography. Abdominal aorta injection: renal artery stenosis.

**Figure 5:**
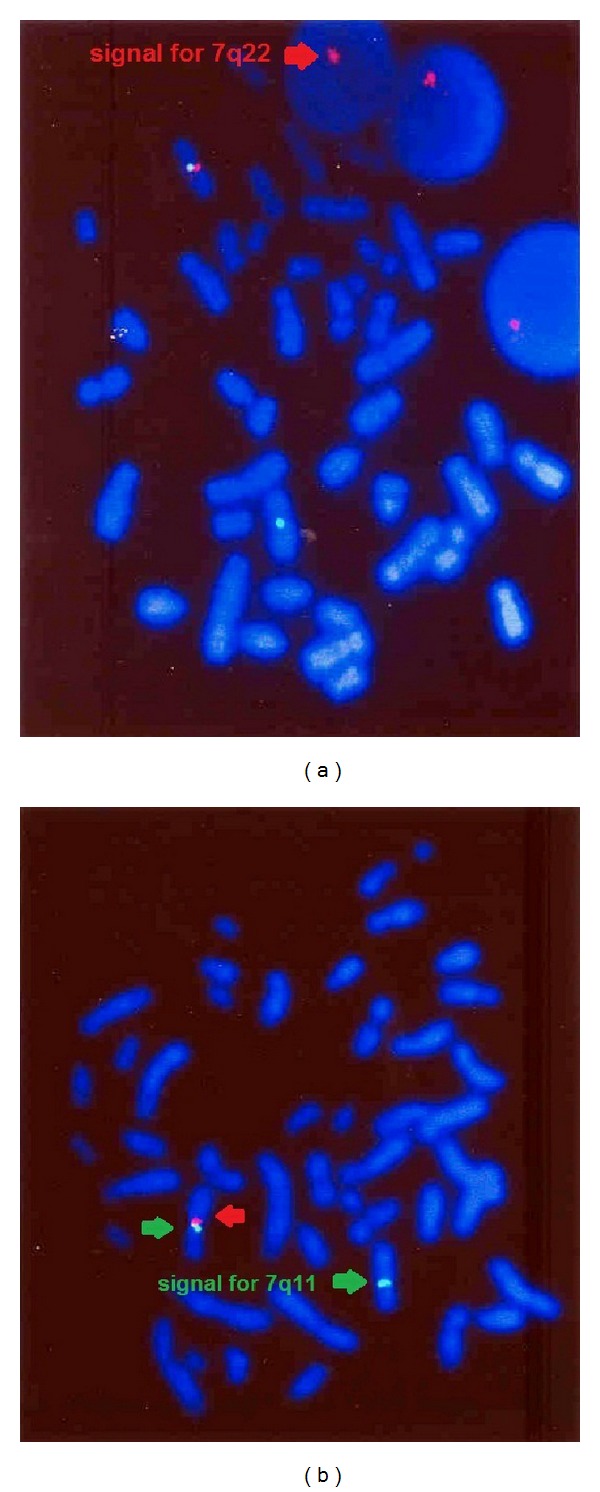
FISH report: signal for 7q11 is labeled in green and 7q22 region is labeled in red. Fluorescent in situ hybridization was performed using cytocell Williams-Beuren region probe. The Williams-Beuren region probe is optimized to detect copy numbers of the ELN gene region at 7q11.2. The 7q22 region-specific DNA probe at 7q22 is included as control probe, where the signal for 7q11 is labeled in green and 7q22 region is labeled in red. The patient was found to have two chromosomes 7, one of which showed no signals corresponding to the test probes diagnostic Williams syndrome. Conclusion: 46.XX, ish del (7q11.2) (ELN X1) (7q22 X2) ELN deletion compatible with Williams' syndrome.

This paper present a 5-year-old girl that referred to pediatric cardiologist because of heart murmurs. She had a systolic heart murmur (2-3/6) in her right upper sternal border with radiation to right cervical region. The patient had a typical face with bulge forehead (Figure 1), and also she had a speech problem; although she had a good auditory rote memory and could follow the orders, she could not normally say the words and sentences. Echocardiography (GE vivid S5) with probe 5 MHz revealed a mild supraaortic valve stenosis and mild supravalvar and peripheral pulmonary stenosis; then angiography was done and showed: left ventricle injection: mild supraaortic stenosis (Figure 2), in pulmonary artery injection: mild multiple peripheral pulmonary stenosis (Figure 3), and in abdominal aorta injection: bilateral renal arteries stenosis (Figure 4). Left ventricle pressure on cardiac catheterization was 150/0–10 mmHg, and blood pressure in aorta after supravalvar stenosis was 120/60 (80) mmHg. After that, with suspicious to Williams' syndrome, the patient was referred for genetic evaluation. Fluorescent in situ hybridization was performed (Figure 5), and the result was 46.XX, ish del (7q11.2) (ELN X1) (7q22 X2) ELN deletion compatible with Williams' syndrome. The diagnosis of Williams' syndrome was made, and the patient is under observation. In our patient, hemodynamic was normal and there was no sign of cardiac hypertrophy or heart failure; thus the patient is under observation and recurrent echocardiography, and if stenosis gets worse in followup, then surgery may be necessary.

## 3. Discussion

This paper is about a case of Williams' syndrome that referred to pediatric heart center with heart murmur, cognitive deficit, and typical faces. For evaluating a heart murmur, echocardiography was done and showed a mild supraaortic valve stenosis and mild supravalvar and peripheral pulmonary stenosis. Echocardiography with Doppler is highly sensitive in the diagnosis of supravalvular aortic stenosis, but it does not completely delineate the anatomy. Cardiac catheterization with angiography can define the anatomy and physiology in detail, but magnetic resonance imaging [8] and multidetector CT-scanning are less invasive, lower-risk methods of accurately defining the anatomy. The risk of cardiac catheterization is significant [9, 10]; so angiography was done for this patient and revealed: mild multiple peripheral pulmonary stenosis, mild supraaortic stenosis, and bilateral renal arteries stenosis.

Stenosis of the pulmonary arteries, isolated or in association with other cardiac defects, occurs in 2-3% of congenital heart diseases [11]. The association of supravalvar aortic stenosis, multiple peripheral pulmonary artery stenosis, mental retardation, and peculiar facies has been described as Williams' syndrome. Peripheral pulmonary artery stenosis is also associated with the Noonan syndrome, the Alagille syndrome, cutis laxa, the Ehler-Danlos syndrome, and the Silver-Russell syndrome [12]. Alagille syndrome is defined as the presence of bile duct paucity on liver biopsy in conjunction with three of the following findings: cholestasis, CHDs, skeletal or ocular abnormalities, or typical facial features [13, 14]. The Noonan syndrome's diagnosis is based on the clinical evaluations and it is characterized by hypertelorism, ptosis, short stature, and CHDs [15, 16]. Our patient had peripheral pulmonary artery stenosis and typical facial feature, but she did not have other signs of these syndromes, and in addition, she had a supravalvular aortic stenosis which is not seen in those syndromes except Williams' syndrome.

Supravalvular aortic stenosis, estimated to occur in approximately 1 of 25,000 live births [17] and accounting for approximately 0.5% of congenital heart diseases cases, but only 30–50% of patients with supravalvular aortic stenosis have Williams-Beuren syndrome [18, 19] and about 20% of cases are familial but without other feature of Williams-Beuren syndrome, and the remaining cases, about half, appear to be sporadic. In our patient, supravalvular aortic stenosis was not isolated and she had other clinical features of Williams' syndrome: peripheral pulmonary artery stenosis, elfin facies, and cognitive deficits.

Most patients with supravalvular aortic stenosis are diagnosed during evaluation of an asymptomatic heart murmur [9, 20]. Systolic murmur is similar to valvular aortic stenosis, most prominent at upper right sternal border with radiation into the suprasternal notch and into the neck. Blood pressure in the right arm is frequently higher than the left arm because of Coanda's effect [21]. This patient's diagnosis also was find out during evaluation of heart murmur, and we suggest a more attention for evaluating heart murmur in childhood period especially when the patient has abnormal facial features or mental problem.

If significant supravalvar aortic stenosis is left untreated, cardiac hypertrophy followed by cardiac failure is probable [22]. Although balloon dilation [23] and stent treatment [24] of supravalvular aortic stenosis have been reported, the close proximity to the aortic valve and coronary artery orifices are significant obstacles, and currently operation is the treatment of choice. The rate of restenosis after the operation with current surgical techniques is very low. Indications for surgery are not clearly defined, and also long-term outcome after surgical repair of supravalvular aortic stenosis is not known. Certainly the presence of symptoms warrants prompt consideration for surgery [9, 25]. In this case, there was a mild supravalvular aortic stenosis and there were no signs of cardiac hypertrophy or heart failure; thus surgery was not necessary. Aortic stenosis is followed by recurrent echocardiography and if stenosis gets worse in followup, then surgery may be unavoidable. Also the patient's hemodynamic is normal, and if blood pressure get rise due to renal artery stenosis, then renal artery stenting is a choice of treatment.

## 4. Conclusion

According to primary symptoms, paraclinical evaluations, and clinical finding such as elfin faces, neurocognitive deficit, and congenital heart defect, Williams' syndrome is the first diagnose. We suggest a more attention for evaluating heart murmur in childhood period especially when the patient has abnormal facial features or mental problem.
